# *Staphylococcus aureus* isolates from children with clinically differentiated osteomyelitis exhibit distinct transcriptomic signatures

**DOI:** 10.1371/journal.pone.0288758

**Published:** 2023-08-10

**Authors:** Ahmad A. Hachem, Laura M. Filkins, Yared H. Kidane, Prithvi Raj, Naureen G. Tareen, Carlos A. Arana, Gowrishankar Muthukrishnan, Lawson A. Copley

**Affiliations:** 1 Department of Pediatrics, University of Florida College of Medicine –Jacksonville, Jacksonville, FL, United States of America; 2 Department of Microbiology, University of Texas Southwestern, Children’s Health System of Texas, Dallas, TX, United States of America; 3 Center for Pediatric Bone Biology and Translational Research, Scottish Rite for Children, Dallas, TX, United States of America; 4 Microbiome Research Laboratory, University of Texas Southwestern, Dallas, TX, United States of America; 5 Department of Pediatric Orthopaedic Surgery, Children’s Health System of Texas, Dallas, TX, United States of America; 6 Genomics Core, University of Texas Southwestern, Dallas, TX, United States of America; 7 Center for Musculoskeletal Research, University of Rochester Medical Center, Rochester, NY, United States of America; 8 Department of Pediatric Orthopaedic Surgery, University of Texas Southwestern, Dallas, TX, United States of America; Tribhuvan University, NEPAL

## Abstract

There is substantial genomic heterogeneity among *Staphylococcus aureus* isolates of children with acute hematogenous osteomyelitis (AHO) but transcriptional behavior of clinically differentiated strains has not been previously described. This study evaluates transcriptional activity of *S*. *aureus* isolates of children with AHO that may regulate metabolism, biosynthesis, or virulence during bacterial growth and pathogenesis. *In vitro* growth kinetics were compared between three *S*. *aureus* clinical isolates from children with AHO who had mild, moderate, and severe illness. Total RNA sequencing was performed for each isolate at six separate time points throughout the logarithmic phase of growth. The NASA RNA-Sequencing Consensus Pipeline was used to identify differentially expressed genes allowing for 54 comparisons between the three isolates during growth. Gene Ontology (GO) and Kyoto Encyclopedia of Genes and Genomes (KEGG) enrichment pathways were used to evaluate transcriptional variation in metabolism, biosynthesis pathways and virulence potential of the isolates. The *S*. *aureus* isolates demonstrated differing growth kinetics under standardized conditions with the mild isolate having higher optical densities with earlier and higher peak rates of growth than that of the other isolates (p<0.001). Enrichment pathway analysis established distinct transcriptional signatures according to both sampling time and clinical severity. Moderate and severe isolates demonstrated pathways of bacterial invasion, *S*. *aureus* infection, quorum sensing and two component systems. In comparison, the mild strain favored biosynthesis and metabolism. These findings suggest that transcriptional regulation during the growth of *S*. *aureus* may impact the pathogenetic mechanisms involved in the progression of severity of illness in childhood osteomyelitis. The clinical isolates studied demonstrated a tradeoff between growth and virulence. Further investigation is needed to evaluate these transcriptional pathways in an animal model or during active clinical infections of children with AHO.

## Introduction

Children with acute hematogenous osteomyelitis (AHO) caused by *Staphylococcus aureus* demonstrate a wide range of illnesses, ranging from mild to severe [1,2]. Some children have short hospitalizations without surgery, while others develop septic shock and have prolonged bacteremia, requiring intensive care and surgical source control during prolonged hospitalizations [1,2]. The underlying pathogenetic mechanisms which lead to this wide spectrum of clinical phenotypes of AHO have yet to be established with respect to either the host or pathogen. Previous studies have evaluated the host gene expression from blood samples of children with *S*. *aureus* AHO and observed up-regulation of innate immunity (mainly neutrophil activity) and down regulation of adaptive immunity (T cells, B cells, and NK cells) during the period of acute infection [3–5]. However, it is unclear how these activities potentiate differentiation toward mild or severe illness. From the perspective of the pathogen, a high-resolution transcriptomic analysis of *S*. *aureus* in a mouse hematogenous model identified differentially expressed genes in acute and chronic osteomyelitis [6]. Genes that mediate metabolic adaptation, immune evasion, and replication appear to drive acute osteomyelitis. Whereas in chronic osteomyelitis the pathogen switches its transcriptional response to a persistence mode driven by nutritional deficiencies [6]. This suggests that transcriptional differences of the pathogen may play an important role in the development and progression of AHO. This theory is further supported by substantial genomic heterogeneity which has been observed among *S*. *aureus* isolates obtained from children with AHO within a single community [7]. A phylogenetic analysis of seventy-one clinical isolates from children with a wide spectrum of clinical phenotypes, ranging from mild to severe, demonstrated correlation of the genetic distances between isolates and the severity of illness scores of the affected children [8]. This study analyzes and demonstrates important differences in the growth kinetics and transcriptional signatures of *S*. *aureus* isolates obtained from children with clinically differentiated AHO.

## Methods

### Bacterial strain selection

This study was conducted following Institutional Review Board (IRB) approval: IRB Number STU 032013–022. The *S*. *aureus* isolates were identified from a repository of clinical strains previously evaluated with whole genome sequencing [7,8]. Organisms were selected from opposite ends of the phylogenetic map to ensure maximum genetic distance between isolates and wide clinical differentiation based on the severity of illness scores of the affected children. Additionally, one pathogen was chosen in the middle of the phylogenetic map which had been obtained from a child with moderate illness to provide an additional comparator within the spectrum of illness severity. The isolates studied included: Methicillin-sensitive *S*. *aureus* (MSSA)-29 (mild AHO), Methicillin-resistant *S*. *aureus* (MRSA)-12 (moderate AHO), and MRSA-9 (severe AHO). The basis for the selection of these differing clinical strains is to initiate a query into the foundational mechanisms of disease of childhood osteomyelitis. It is our hypothesis that clinical severity of illness is driven by the transcriptional signaling introduced by the pathogenic strains of *S*.*aureus* within local communities.

### Growth kinetics study and analysis

The clinical isolates were cultured on Tryptic soy agar with 5% sheep blood for 24 hours at 37°C in ambient air. A pre-inoculum growth culture was prepared by inoculating a single colony into 2 mL of Mueller-Hinton broth (MHB, Thermo Fischer Scientific, Massachusetts, United States; 37°C, with agitation at 225 rpm, and room air humidity) to achieve a target optical density at 600 nm (OD_600_) of 0.5. Based on protocol optimization, 0.5 OD_600_ occurred at about 4 hours for MSSA-29 (mild), 5 hours for MRSA-12 (moderate), and 5.5 hours for MRSA-9 (severe). For growth kinetics and RNA sequencing culture preparation, an aliquot of 0.5 OD_600_ pre-inoculum growth culture was inoculated into 25 mL MHB to a starting OD_600_ of 0.025. Cultures were incubated at 37°C with agitation at 225 rpm and room air humidity. Growth kinetics studies were evaluated by serially removing broth culture aliquots and measuring growth by OD_600_ and CFU/mL every hour, for three separate colonies per strain. A one-way ANOVA, followed by the Tukey method for multiple comparisons, was performed to identify statistically significant differences (p<0.05) of OD_600_ measurements and rates of change for the three isolates throughout the growth cycle.

### RNA isolation, library construction and sequencing

Bacterial RNA isolation was performed from 1.0 mL aliquots of broth culture obtained at 40-minute intervals from 4.5 to 7.8 hours. This time-period correlated with the growth phase intended for transcriptional comparison, with each clinical isolate analyzed in triplicate. RNA-stabilized samples were homogenized in 1 ml of MHB and centrifuged at 13,300 x g for 3 minutes at 4°C to collect the bacterial pellet. RNA was extracted using TRizol reagent (Sigma-Aldrich, United States). The bacterial pellets were then re-suspended in pre-loaded RINO tubes for homogenization of bacterial samples in BBY24M Bullet Blenders® (Next Advance, United States). Total RNA was extracted using the Bacterial Total RNA extraction Kit (Sigma-Aldrich, USA). Quantity and quality of RNA samples were measured by an Agilent Bioanalyzer 2100 (Agilent Technologies, Santa Clara, California, USA). RNA integrity number (RIN) ≥ 9.0 was considered as quality pass for sequencing. RNA sequencing libraries were prepared with Zymo-Seq RiboFree Total RNA Library Kit (R3000) according to the manufacturer’s protocol. Libraries were validated on an Agilent Bioanalyzer 2100. Indexed libraries were equimolarly pooled and sequenced on a SE75 (single-end 75 base pair) Illumina NextSeq550 flow cell. About 20 million sequencing reads were generated for each sample. All sequence data were submitted to NCBI Gene Expression Omnibus (GEO) under the accession number 981186 which is available at the following URL: ID 981186—BioProject—NCBI (nih.gov).

### RNA sequence analysis, GO and KEGG pathway enrichment analysis

The NASA RNA-Sequencing Consensus Pipeline (RCP) was used for the analysis [9]. Briefly, the quality of sequence reads was assessed using FastQC/MultiQC. Sequencing reads were mapped to the USA 300 *S*. *aureus* reference strain genome of UTSW55 (NCBI GenBank Reference NZ_CP013231.1) [10] using STAR aligner. Counts were then quantified using RSEM. Following this, normalization of counts and identification of differentially expressed genes (DEGs) were performed using DESeq2 [10]. Significant DEGs were selected using fold-change >2 and adjusted p-value <0.05.

Global transcriptional differences between isolates over the progression of growth were evaluated with Principal Component Analysis (PCA) and volcano plots which were generated for three contrasts: MRSA-9 vs MSSA-29 (severe vs mild), MRSA-12 vs MSSA-29 (moderate vs mild), and MRSA-9 vs. MRSA-12 (severe vs moderate). Enrichment of Gene Ontology (GO) and Kyoto Encyclopedia of Genes and Genomes (KEGG) pathways was performed using the Gene Set Enrichment Analysis technique [11,12] with the goal to identify and evaluate similarities and differences of perturbed pathways among the various virulence levels and timepoints. A P-value of < 0.05, adjusted by false discovery rate (FDR), was considered to have statistical significance to achieve significant enrichment.

## Results

### Bacterial growth kinetics

**Fig 1:**
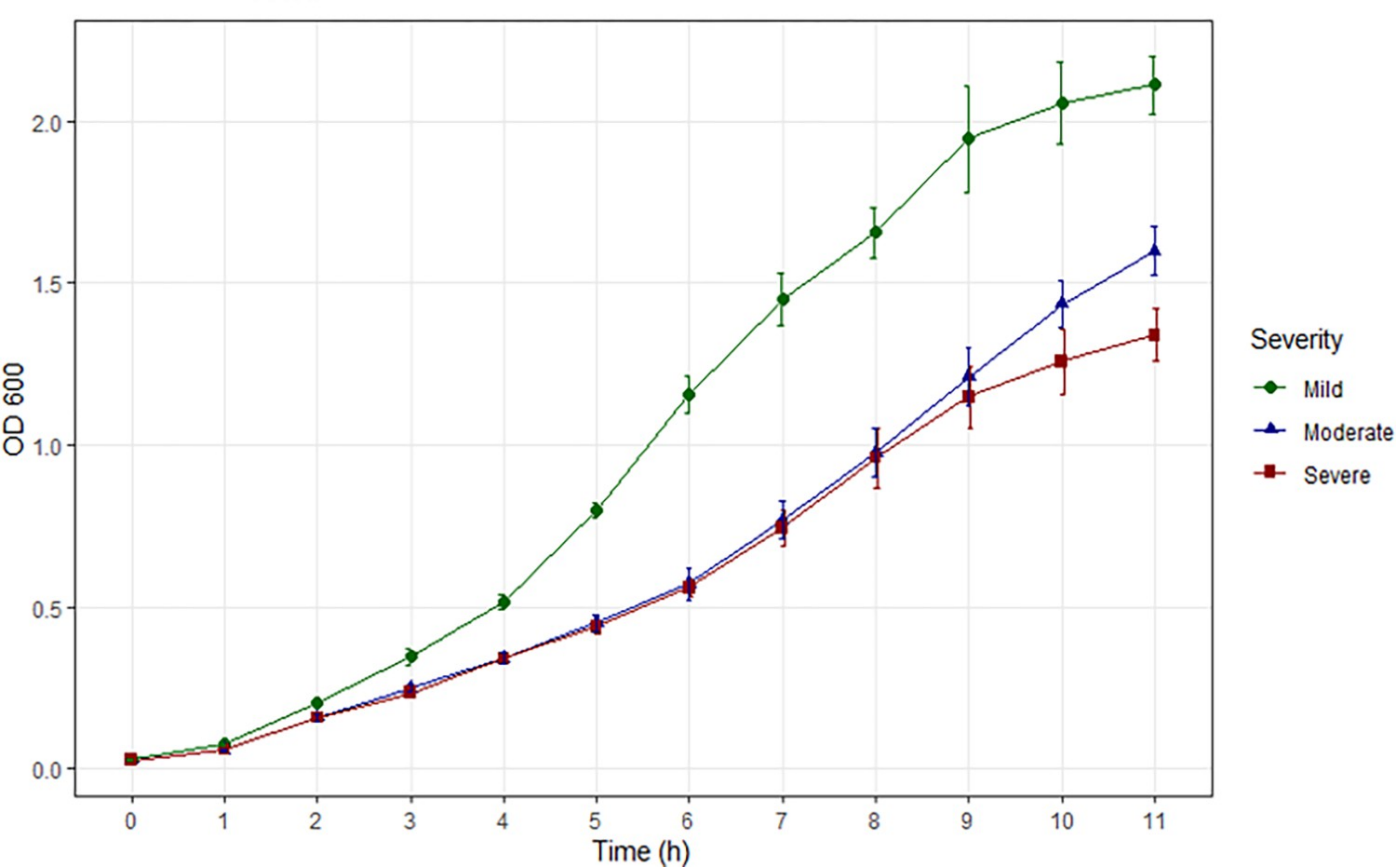
Comparative growth of the three clinical isolates (MSSA29 –mild; MRSA12 –moderate; MRSA9 –severe) by OD_600_ throughout the growth cycle after standardized inoculation in MHB at time zero (OD_600_ = 0.025). Growth curves suggest that the RNA sequencing time window from 4.5 to 7.8 hours was inclusive of the logarithmic phase of growth.

The three study isolates demonstrated differing growth kinetics based on OD_600_ (Fig 1 and Table 1). The mild strain (MSSA-29) had significantly higher OD_600_ measurements (p<0.001) at all time points than that of either the moderate (MRSA-12) or severe (MRSA-9) strain. The moderate strain demonstrated a trend of higher OD_600_ at 10 hours and significantly greater (p = 0.0013) than the severe isolate at 11 hours of growth. The mild strain reached its peak growth rate in 5 to 6 hours after inoculation, the moderate strain in 8 to 9 hours and the severe strain in 7 to 8 hours.

**Table 1:** Comparison of bacterial illness severity by OD_600_ growth over time.

Time Points	3 (Severe)			p-value			Tukey-test						
	N	Mean	SD	N	Mean	SD	N	Mean	SD		2 vs.1	3 vs.1	3 vs.2
0h	3	**0.027**	0.001	3	**0.026**	0.003	3	**0.026**	0.001	0.4867			
1h	3	**0.074**	0.003	3	**0.056**	0.005	3	**0.059**	0.006	0.0000	0.0000	0.0002	0.5260
2h	3	**0.200**	0.010	3	**0.158**	0.013	3	**0.157**	0.004	0.0000	0.0000	0.0000	0.9696
3h	3	**0.345**	0.030	3	**0.248**	0.013	3	**0.233**	0.010	0.0000	0.0000	0.0000	0.4153
4h	3	**0.515**	0.029	3	**0.340**	0.020	3	**0.340**	0.019	0.0000	0.0000	0.0000	1.0000
5h	3	**0.797**	0.031	3	**0.448**	0.035	3	**0.438**	0.025	0.0000	0.0000	0.0000	0.8425
6h	3	**1.157**	0.072	3	**0.570**	0.062	3	**0.558**	0.032	0.0000	0.0000	0.0000	0.9353
7h	3	**1.450**	0.103	3	**0.767**	0.072	3	**0.743**	0.069	0.0000	0.0000	0.0000	0.8784
8h	3	**1.657**	0.098	3	**0.975**	0.093	3	**0.958**	0.113	0.0000	0.0000	0.0000	0.9565
9h	3	**1.943**	0.203	3	**1.210**	0.112	3	**1.147**	0.120	0.0000	0.0000	0.0000	0.7510
10h	3	**2.057**	0.159	3	**1.435**	0.093	3	**1.257**	0.125	0.0000	0.0000	0.0000	0.0726
11h	3	**2.112**	0.111	3	**1.598**	0.093	3	**1.338**	0.100	0.0000	0.0000	0.0000	0.0013

### Cellular aggregation during growth

Polysaccharide intercellular adhesin [13] locus (*ica*ABCD), fibronectin-binding protein (*fnb*A), fibrinogen-binding protein (*fib*), and Protein A (*spa*) comparisons demonstrated significantly differential gene expression between isolates, particularly between the moderate or severe strains when compared with the mild strain (Table 2). Upregulation of *fib*, *fnb*, and *ica* along with downregulation of spa occurred over the first four time points. The severe and moderate strains showed upregulation of fibrinogen binding of MRSA-9 until the last two time periods of growth when *fib*, *fnb*A, and *ica* became downregulated while spa activity increased. This late (T8-T9) transcriptional pattern was similarly found between MRSA-9 and MSSA-29.

**Table 2:** MRSA-9 (severe) versus MSSA-29 (mild) differentially expressed genes responsible for intercellular adhesion and aggregation.

	**T4 (4.8h)**		**T5 (5.2h)**		**T6 (5.8h)**		**T7 (6.5h)**		**T8 (7.2h)**		**T9 (7.8h)**	
**Gene Symbol**	log2FC	p_adj	log2FC	p_adj	log2FC	p_adj	log2FC	p_adj	log2FC	p_adj	log2FC	p_adj
**fib**	3.11	1.53931E-55	4.10	2.8963E-93	3.39	1.50761E-65	3.37	3.15864E-64	-3.02	1.96779E-52	-2.90	9.71605E-48
**fnbA**	4.15	7.47507E-37	5.34	6.51483E-60	3.83	1.42637E-31	4.65	5.00885E-46	-3.48	1.99006E-26	-3.50	1.57732E-26
**icaA**	0.92	0.057080722	0.80	0.108160989	1.39	0.002858087	2.16	4.57408E-06	-1.59	0.000649323	-0.19	0.720617313
**icaB**	0.98	6.95685E-05	1.05	3.53082E-05	1.45	5.97653E-09	2.73	3.01357E-21	-1.77	1.55832E-11	-0.47	0.087654855
**icaC**	0.63	0.005271822	0.28	0.253899095	0.48	0.035865195	1.99	1.02524E-18	-1.25	1.81123E-08	-0.19	0.454503645
**icaD**	1.22	0.00955073	1.62	0.00127201	1.68	0.000465894	2.59	1.75372E-05	-2.11	5.4712E-05	-0.64	0.239283767
**spa**	-2.43	5.49336E-18	-2.89	7.05928E-25	-2.94	1.10698E-25	-1.37	1.34975E-06	2.02	5.56157E-13	1.02	0.000406261
	**MRSA-12 (Moderate) Versus MSSA-29 (Mild)**											
	**T4 (4.8h)**		**T5 (5.2h)**		**T6 (5.8h)**		**T7 (6.5h)**		**T8 (7.2h)**		**T9 (7.8h)**	
**Gene Symbol**	log2FC	p_adj	log2FC	p_adj	log2FC	p_adj	log2FC	p_adj	log2FC	p_adj	log2FC	p_adj
**fib**	2.22	1.25762E-28	2.93	9.71791E-48	2.26	2.51344E-29	1.92	1.8854E-21	-1.28	3.03191E-09	-1.01	9.83107E-06
**fnbA**	3.58	1.30403E-27	4.51	9.55062E-43	2.76	7.48041E-17	3.13	1.89499E-21	-1.31	0.001056568	-1.14	0.008780448
**icaA**	1.01	0.032399079	0.77	0.130481343	0.62	0.227976701	2.65	1.59026E-08	-0.54	0.512500445	-0.18	0.917787204
**icaB**	0.94	0.000140727	1.12	1.0271E-05	1.25	9.86491E-07	2.58	5.141E-19	-0.54	0.118356651	-0.15	0.848408189
**icaC**	0.42	0.065496148	0.28	0.261554767	0.17	0.511267382	1.63	9.42242E-13	-0.36	0.289071105	-0.23	0.622315557
**icaD**	0.88	0.068299476	1.85	0.000218461	1.33	0.008547546	3.10	2.18212E-07	-0.53	0.524806768	-0.15	0.932996066
**spa**	-3.14	3.11194E-29	-3.19	7.76324E-30	-3.43	3.38778E-34	-1.72	1.08171E-09	-0.59	0.151761468	-1.10	0.002000207
	**MRSA-9 (Severe) Versus MRSA-12 (Moderate)**											
	**T4 (4.8h)**		**T5 (5.2h)**		**T6 (5.8h)**		**T7 (6.5h)**		**T8 (7.2h)**		**T9 (7.8h)**	
**Gene Symbol**	log2FC	p_adj	log2FC	p_adj	log2FC	p_adj	log2FC	p_adj	log2FC	p_adj	log2FC	p_adj
**fib**	0.90	0.000334602	1.17	1.12471E-07	1.13	3.19087E-07	1.449	7.13873E-12	-1.74	3.6498E-18	-1.88	1.10933E-20
**fnbA**	0.58	0.598858134	0.84	0.069265497	1.08	0.011017827	1.517	0.000110772	-2.18	4.25191E-11	-2.36	1.02994E-12
**icaA**	-0.09	1	0.03	NA	0.77	0.216841521	-0.490	0.462355759	-1.05	0.027808394	-0.01	0.981012915
**icaB**	0.04	1	-0.07	NA	0.20	0.583686049	0.147	0.738166879	-1.23	4.87392E-06	-0.32	0.273830003
**icaC**	0.20	0.913155977	0.00	0.998671345	0.31	0.320029937	0.367	0.204377139	-0.89	7.90801E-05	0.04	0.887961289
**icaD**	0.34	0.9552403	-0.23	NA	0.35	0.607961439	-0.508	0.426825701	-1.58	0.003217788	-0.49	0.393070975
**spa**	0.72	0.265396205	0.30	0.546515262	0.49	0.215866432	0.353	0.400396411	2.61	1.17539E-20	2.12	6.36311E-14
		Significantly downregulated										
		Significantly upregulated										
		No significant difference										

### Differentially expressed genes by severity and time

**Fig 2:**
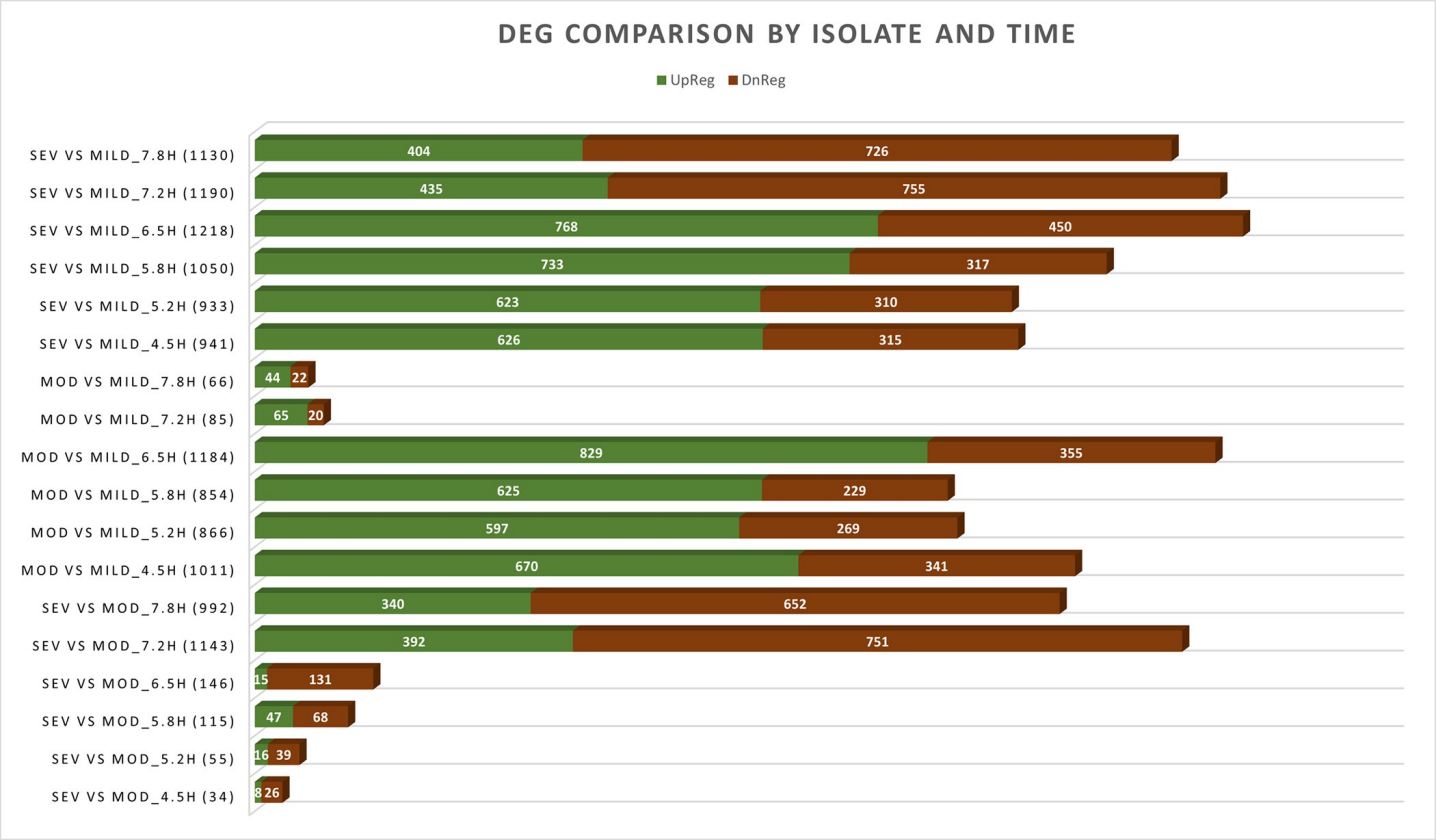
Number of Differentially Expressed Genes (DEGs) according to locus ID compared by isolate and time point of growth. Upregulated genes are displayed in green and downregulated genes in red.

DEG quantification was performed at each timepoint for each comparator group which established the distribution of up-regulation versus down-regulation based on locus IDs (Figs 2 and S1). Two distinct transcriptional signatures were observed. MRSA-9 and MRSA-12 were differentiated from MSSA-29 during the early time points from 4.5 to 6.5 hours when the greatest number of DEGs occurred, with a range from 933 to 1,218 DEGs per time point for the severe to mild comparison, and 854 to 1,184 DEGs per time point for the moderate to mild comparison. At later time points, a second transcriptional signature emerges, distinguishing the moderate (MRSA-12) from the severe (MRSA-9) strains with an increase in DEGs (1,143 DEGs at 7.2 hours and 992 DEGs at 7.8 hours). During that same time period, the transcriptional differences between the moderate and mild isolates diminish as is demonstrated by the sharp decrease in DEGs (85 at 7.2 hours and 66 at 7.8 hours).

**Fig 3:**
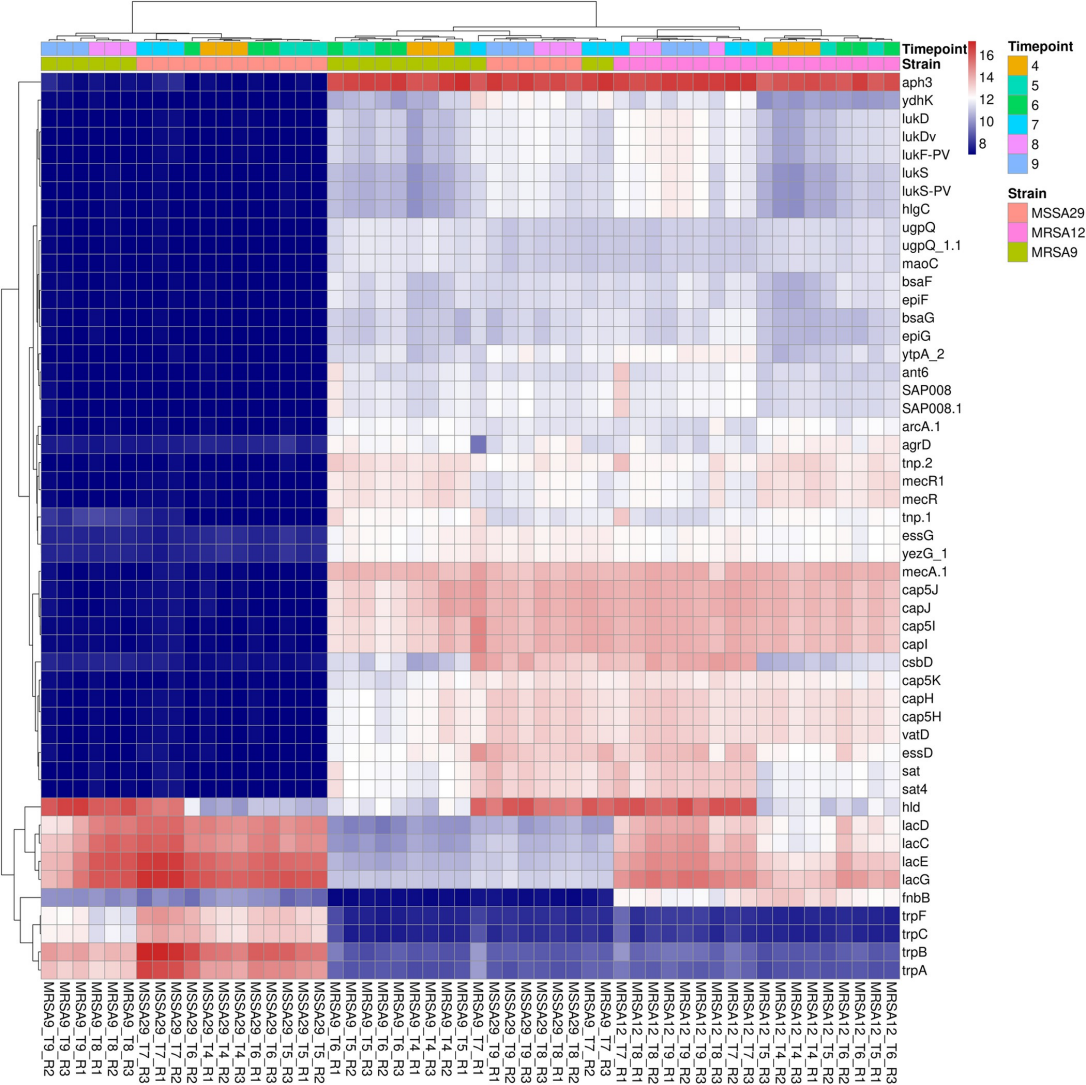
Heat map of top 50 genes with significant variation between strains and time points. Genes of interest include leukocidins (lukD, lukDv, LukF-PV, lukS lukS-PV), hemolysins (hlgC), virulence regulation (agrD, essG, essD), adhesins (fnbB), and iron metabolism (cap) which display significant downregulation for MSSA-29 at time points T4 to T7 (early) and MRSA-9 at time points T8-T9 (late). Lactose metabolism (lac) is highly upregulated for MSSA-29 during the early time period and downregulated late. The converse is true for MRSA-9 which downregulates lactose metabolism early and upregulates this transcription late. The R letters on the x-axis indicated the replicate number (e.g. R1 is indicative of Replicate 1).

A heat map of the top 50 up- or down-regulated genes (Fig 3) provides greater detail of differences between transcriptional signatures of the isolates. The leukocidins, hemolysins, adhesins and iron metabolism genes are down-regulated for the mild strain from 4.5 to 6.5 hours. These same genes become down-regulated for the severe strain at 7.2 and 7.8 hours. Whereas the genes for lactose metabolism (lac) are highly upregulated in the mild strain during the first four time points and upregulated in the severe strain during the final two time points.

### GO and KEGG pathway enrichment analysis

**Fig 4:**
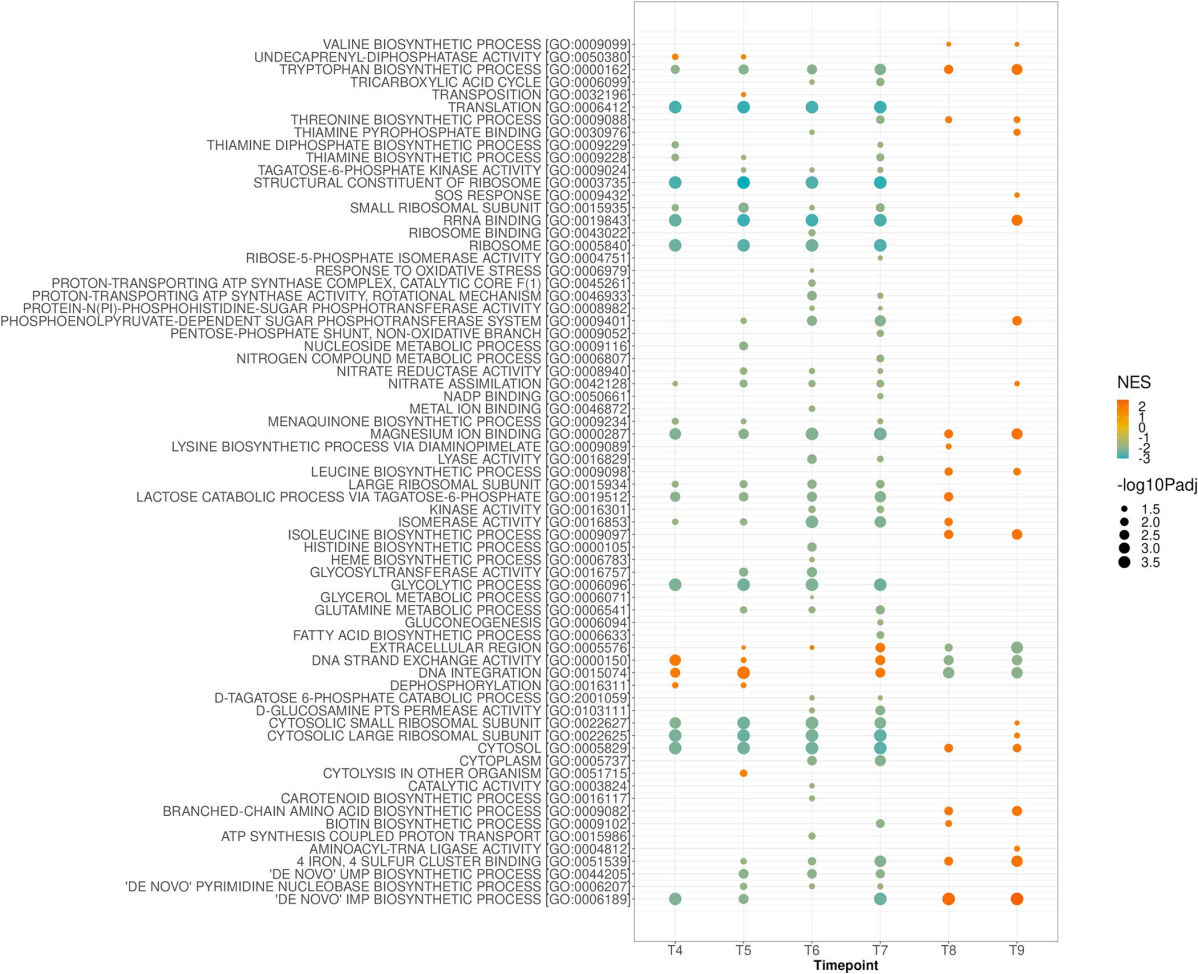
Gene Ontology (GO) analysis of MRSA-9 (severe) versus MSSA-29 (mild) strains showing downregulation of rRNA and ribosome binding, biosynthetic and metabolic pathways and upregulation of DNA strand exchange and DNA integration of MRSA-9 relative to MSSA-29 until late in the growth process when the opposite behaviors are expressed.

GO pathway analysis illustrates functional differences between isolates. We observed down-regulation of biosynthetic, glycolytic, and metabolic processes demonstrated in severe strain as compared to the mild strain. Conversely, upregulated DNA strand exchange and DNA integration was observed over the first four time points of growth in the severe strain (Fig 4). The GO pathway analysis of the moderate versus mild isolates demonstrated similar pattern of transcription to that of the severe versus mild comparison (S1 Fig). However, when the moderate and severe strains are compared, downregulation of metabolic and biosynthetic activity was observed in severe strain, until late in the growth process (T8 and T9) (S2 Fig).

**Fig 5:**
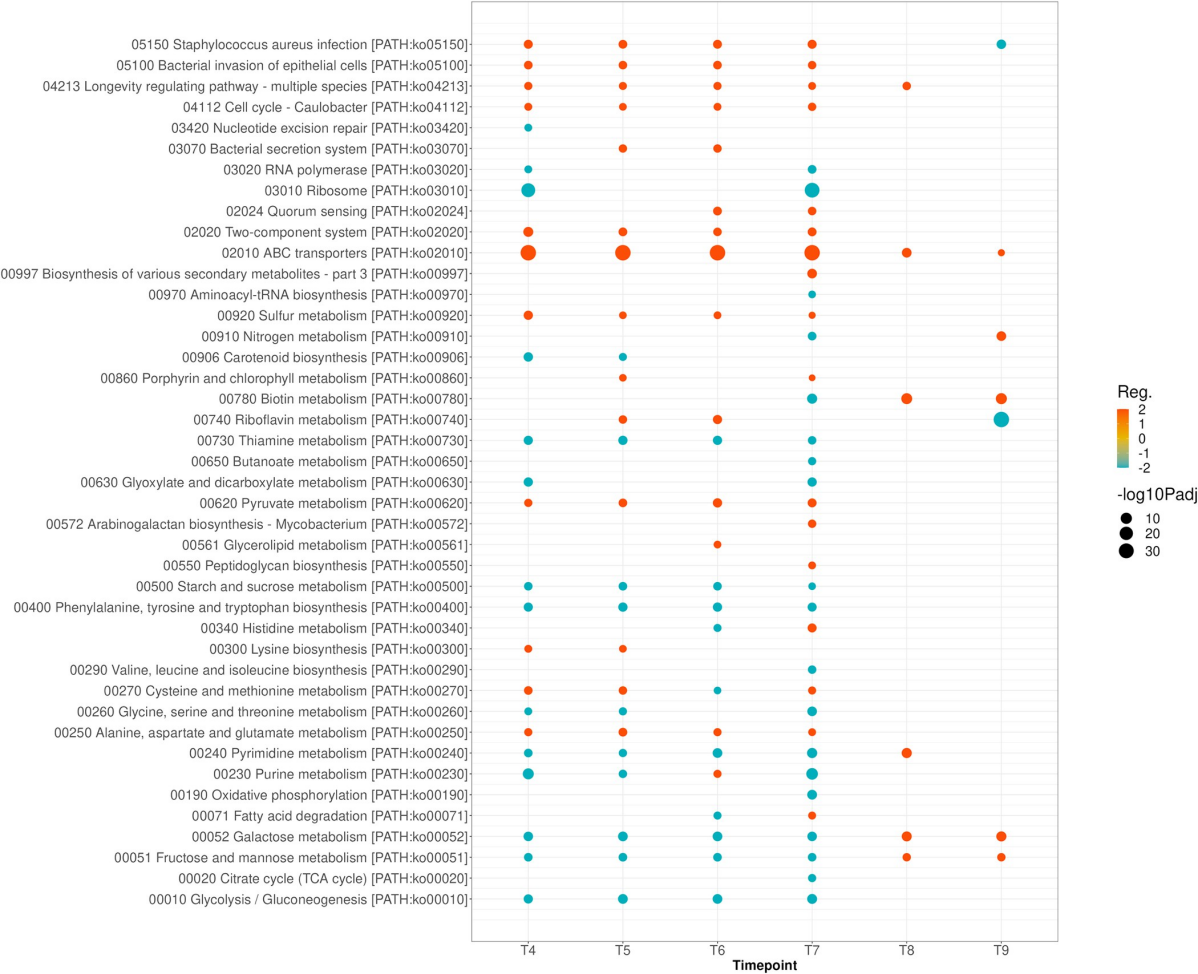
KEGG Enrichment Pathway Analysis of MRSA-9 (severe) versus MSSA-29 (mild) isolates showing upregulation of SA infection, bacterial invasion, two component systems and ABC transporters and downregulation of biosynthesis and metabolism of MRSA-9 relative to MSSA-29. During the last two time points of growth, the transcriptional differences change with MRSA-9 favoring biosynthesis and metabolism pathways relative to MSSA-29. Reg.: Regulation.

KEGG pathway enrichment analysis showed significant upregulation of SA infection, bacterial invasion, longevity regulating pathways, cell cycle, two component systems, alanine, aspartate and glutamate metabolism, and ABC transporters of the severe strain relative to the mild strain (Fig 5). Whereas functions of metabolism and biosynthesis, including glucose, fructose, galactose, and glycolysis were downregulated in the severe strain. In a similar manner to what was depicted with GO analysis, these changes occurred during the last two periods of growth. KEGG pathway enrichment analysis of moderate to mild and moderate to severe strains are provided separately in supplemental materials (S3 and S4 Figs). Of these comparisons, the differences between severe and moderate strains are minimal during the first four time points. During the last two time points, the severe strain demonstrates upregulated metabolic and biosynthesis pathways and downregulated SA infection, bacterial invasion, and ABC transporter activities relative to the moderate strain.

## Discussion

The clinical spectrum of *S*. *aureus* osteomyelitis in growing children ranges in severity from mild, with rapid resolution, to severe, with potential for serious long-term adverse outcomes [14]. Although the *S*. *aureus* genome is comprised of at least 200 virulence genes, the pathogenetic mechanisms which potentiate virulence and lead to clinical phenotype differentiation, are poorly understood [15–18]. Panton-Valentine Leucocidin (PVL) presence among *S*. *aureus* strains in osteomyelitis has been associated with longer duration of infection and greater inflammatory response [19]. However, it has been difficult to establish a definite connection between a specific virulence gene, such as PVL and clinical disease manifestations given the redundancy of two-component systems and downstream toxins purportedly responsible for the virulence behavior of *S*. *aureus* [20]. Previous work has demonstrated genomic heterogeneity among clinical isolates obtained from 71 children with *S*. *aureus* AHO which discovered an association between the phylogenetic interconnection of the pathogens and the severity of illness of the affected children. Isolates associated with mild and severe illness were on opposite ends of the phylogenetic map, whereas isolates retrieved from moderately sick children gathered near the center. This suggested the likelihood of bacterial genetic influence upon the clinical manifestations of this disease. To our knowledge, this is the first study to explore transcriptional pathways of *S*. *aureus* strains obtained from children with well-differentiated clinical disease based on severity of illness. The findings of this study confirm that growth kinetics and transcriptional activity vary among clinical pathogens obtained from children with well-differentiated disease.

With the emergence of high-throughput technologies of next generation sequencing, transcriptomics, and metabolic network analysis, investigators are increasingly able to explore systems of bacterial metabolic, biosynthetic, and virulence behaviors [21–24]. One group evaluated the pan-genome of *S*. *aureus* using sequence data from 64 strains to identify the core genome, shared by all strains of *S*. *aureus*, the accessory genome, present in some, but not all species, and the unique genome, specific to individual isolates [21]. They suggested that *S*. *aureus* strains responsible for severe infections may be identified based on growth capabilities and the presence of specific virulence genes [21], which is consistent with our findings. Another group of investigators explored DEGs of infected patients with osteomyelitis using GO and KEGG enrichment analysis of whole blood samples from affected patients and healthy controls [4]. They found 209 SA infection-related genes (SARGs) and 377 osteomyelitis-related genes (OMRGs) which differentiated infected patients from healthy controls [4]. However, there was no attempt to differentiate clinical phenotypes by severity or explore the underlying pathogenetic mechanisms which may have led to the transcriptional differentiation of the host from healthy control.

Important findings of present study are the tendencies for an isolate which caused mild infection (MSSA-29) to favor growth, metabolism, and biosynthesis, while the moderate (MRSA-12) and severe (MRSA-9) isolates favored infection, invasion, two-component systems, quorum sensing, and the ABC transporters. This is supported by the observation of differing growth kinetics in which the mild strain reached the log phase sooner than the moderate or severe strains and then displayed a higher rate of growth. The growth rate and transcriptional differences observed in this study suggest that *S*. *aureus* tends to make trade-offs between growth and virulence.

Another finding of our study is that the bacterial transcription evolved longitudinally over time in an apparent response to environmental changes. As growth progressed and the nutritional resources of the MHB became limited, the growth kinetics and transcription of the isolates evolved accordingly. This is supported by the GO and KEGG pathway findings in which the transcriptional patterns become truncated or reversed during the late time points of growth. It is also notable that the growth and metabolism functions of MRSA-9 and MRSA-12 were significantly down-regulated relative to MSSA-29 prior to the final two time points when these same functions become significantly upregulated in the moderate and severe strains. Other investigators have analyzed events occurring during exponential and post-exponential growth *in vitro* in *S*. *aureus*, showing that virulence gene expression occurs during the growth phase whereas secreted proteins are expressed during the post-exponential phase [19,25,26]. The complexities involved with quorum sensing and transition to alternative transcriptional behavior have been previously studied to demonstrate that *cod*Y acts to inhibit metabolic genes with feedback mechanisms triggered by an excess of isoleucine which resulted in growth inhibition in wild type strains but not in *cod*Y mutants [27]. In our study, *cod*Y was downregulated from T7 to T9 for MRSA-9 relative to MSSA-29.

In the KEGG enrichment pathway analysis the most significantly upregulated function of the severe isolate was that of ABC transporters. This family of membrane proteins participates in ATP-driven transport mechanisms [28]. They hydrolyze ATP, driving a conformational change of transmembrane domains and allow for inward or outward transport of substrates [29,30]. Several ABC transporters have been associated with virulence behavior of *S*. *aureus*, including the Ecs ATP-binding cassette shown to be essential for SA expression of the virulence regulatory protein Rot [31,32]. Other ABC transporters implicated in *S*. *aureus* pathogenesis include *nik*, a nickel transporter and *cnt*, a nickel/cobalt transporter [31,32]. Our data on ABC transporter gene expression in severe strain is consistent with this literature. Another KEGG pathway difference between the moderate and mild strains was that of *Staphylococcus aureus* infection. *S*. *aureus* is known to express several virulence pathways to evade the host immune system, liberate free iron, adhere to bone or collagen and establish infection [31,33,34]. One virulence mechanism includes the modulation of cationic antimicrobial peptides by increasing the positive charge of the cytoplasmic membrane. The two-component system *gra*RS couples an efflux pump (*vra*G) to an ATPase (*vra*F) that engages in sensing human defensins [35]. Once these positively charged defensins are detected, *S*. *aureus* induces an increase in the surface positive charge and decreases the effective killing of those defensins [35]. Other genes involved in this functional pathway include those encoding for surface proteins like clumping factor B (*clf*B), iron-regulated surface determinant (*isd*B) promote adhesion *in vitro* [36,37]. Another surface protein includes the *S*. *aureus* surface protein (*sas*G) which is known to mask SA microbial surface components recognizing the adhesive matrix molecules (MSCRAMMs) binding to their ligand and promoting biofilm formation [35,38].

Limitations of this study include the inability to extrapolate results to the human condition of childhood osteomyelitis because of the *in vitro* methodology. The transcriptional differences observed occurred in rich media under standardized growth conditions. Therefore, it is not possible to predict how these isolates would behave in constrained media, an *in vivo* model, or a human host. However, the results of this study do confirm that organism-specific transcriptional behavior exists among well-differentiated *S*. *aureus* clinical isolates. It is therefore plausible that these organism-specific, unique transcriptional signatures would play a role in the clinical phenotype differentiation of children with AHO.

Another study limitation is the comparison of isolates which are growing at different rates through the exponential phase. The decision to isolate RNA longitudinally over time rather than in a manner targeted to specific OD_600_ values was intentional. It was discovered during growth curve optimization how challenging it would be to match optical densities. Performing a comparative analysis between isolates at one specific OD was beyond the scope of this study. The data derived from optical density or CFU/mL values does not necessarily reflect the state of active organism growth, given that aggregation may vary substantially between isolates, as was shown in this study. This is additionally confirmed by the finding that the OD_600_ of the isolates had only one intersection, approximately 0.75 at T4(4.5h) for MSSA-29, T7(6.5h) for MRSA-12 and T8(7.2h) for MRSA-9. At that intersection of optical densities, there were substantial differences of CFU/mL values with 1.79E+10 for MSSA-29, 3.78E+10 for MRSA-12, and 5.11E+10 for MRSA-9. Investigators have previously shown that *S*. *aureus* has a tendency for aggregate formation to occur early in the growth cycle with 50% of cells assembled into aggregates at OD_600_ of 0.5[38]. Key contributors to this tendency to aggregate and form biofilm include *S*. *aureus* cell surface proteins (*fib* and *fnb*), *spa*, and PIA (*ica*ABCD) [13,39]. The differential expression of these genes identified in this study lend evidence that aggregation potentially alters growth kinetics. It is notable that the growth of MRSA-12 and MRSA-9 did not differ according to CFU/mL or OD_600_ over the six time points of the study. Despite similar growth behavior, these isolates demonstrated unique transcriptional behavior, including that of PIA, fnb and fib during the later stages of growth. Our preliminary growth studies found that MRSA-9 and MRSA-12 significantly diverged at 10 to 11 hours of growth, with the moderate isolate outpacing the severe. The transcriptional distinctions between MRSA-9 and MRSA-12 which occurred at T8 and T9 in this study are premonitory of the kinetic differences that were observed over 11 hours of sustained growth.

While there are numerous DEGs identified in this study, the relative importance of any given pathway to the development or progression of osteomyelitis cannot be ascertained from this data. There are challenges in applying pathway databases of KEGG and GO to a clinical condition that is potentially far more complex that the theoretical network models may be able to depict. Enrichment analysis methods potentially make unrealistic assumptions of statistical independence among genes. Another drawback of pathway enrichment analysis is that these methods ignore genes with no pathway annotations. Ultimately these genes may need to be studied separately in future work.

This study confirms distinct transcriptional profiles among clinical strains of *S*. *aureus in vitro* and invokes consideration that the varied transcriptomes may underly the pathogenetic mechanisms leading to well-differentiated phenotypes of disease among children with AHO. Pathogens leading to severe illness may do so by favoring pathways of virulence at the expense of growth being mediated by two-component systems, *S*. *aureus* infection, quorum sensing, and ABC transporters. By gaining an understanding of the promotors and inhibitors of these pathways it would be possible to conceive of novel therapeutic strategies to interfere with the mechanisms leading to severe illness. Further work is needed to validate the results of this study using an *in vivo* model or samples of infected tissue procured during active infections of children with AHO.

## Supporting information

S1 Fig Gene Ontology (GO) analysis of MRSA-12 (moderate) versus MSSA-29 (mild) isolates showing downregulation of translation, rRNA binding, biosynthetic and metabolic pathways and Upregulation of DNA integration and DNA strand exchange activity compared to MSSA-29 mainly until the last two time points where we notice the opposite behaviors are expressed. NES: Normalized Enrichment Score.
(TIF)

S2 Fig Gene Ontology (GO) analysis of MRSA-9 (severe) versus MRSA-12 (moderate) strains showing down-regulation of biological processes such as translation and rRNA binding, biosynthetic processes like glycolysis, ion and metal absorption. In the later stages, the most notable aspect was down-regulation of DNA Integration but up-regulation of several biosynthetic pathways most prominently amino-acids (leucine, tryptophan and threonine) of MRSA-9 versus MRSA-12. NES: Normalized Enrichment Score.
(TIF)

S3 Fig KEGG Enrichment Pathway Analysis of MRSA-12 (moderate) versus MSSA-29 (mild) isolates showing upregulation of virulence pathways such as ‘SA infection’, ‘Bacterial invasion of epithelial cells’ and downregulation of biosynthetic pathways especially within the earlier phases of growth. Notably the pathway’ ABC transporters’ was constantly upregulated throughout all time points. During the last 2 points, we notice a shift favoring metabolism and biosynthetic bring upregulated in MRSA-12 versus MSSA-29. KEGG: Kyoto Encyclopedia of Genes and Genomes. Reg.: Regulation.
(TIF)

S4 Fig KEGG Enrichment Pathway Analysis of MRSA-9 (severe) versus MRSA-12 (moderate) isolates showing very little differentiation in pathway regulation between the two, especially at the very early time points with down-regulation of ABC transporters, nitrogen and galactose metabolism for MRSA-9. During the last 2 time points, a substantial shift in behavior with up-regulation of most metabolic and biosynthetic pathways and down-regulation of virulence pathways (ABC transporters, SA infection, Bacterial epithelial cells invasion) of MRSA-9 versus MRSA-12. KEGG: Kyoto Encyclopedia of Genes and Genomes. Reg.: Regulation.
(TIF)

S1 File (DOCX)
