# Supplementary figures and images for: Staphylococcus aureus isolates from children with clinically differentiated osteomyelitis exhibit distinct transcriptomic signatures
Source: PLoS One. 2023 Aug 10;18(8):e0288758. doi: 10.1371/journal.pone.0288758 (PMC10414669; doi:10.1371/journal.pone.0288758)

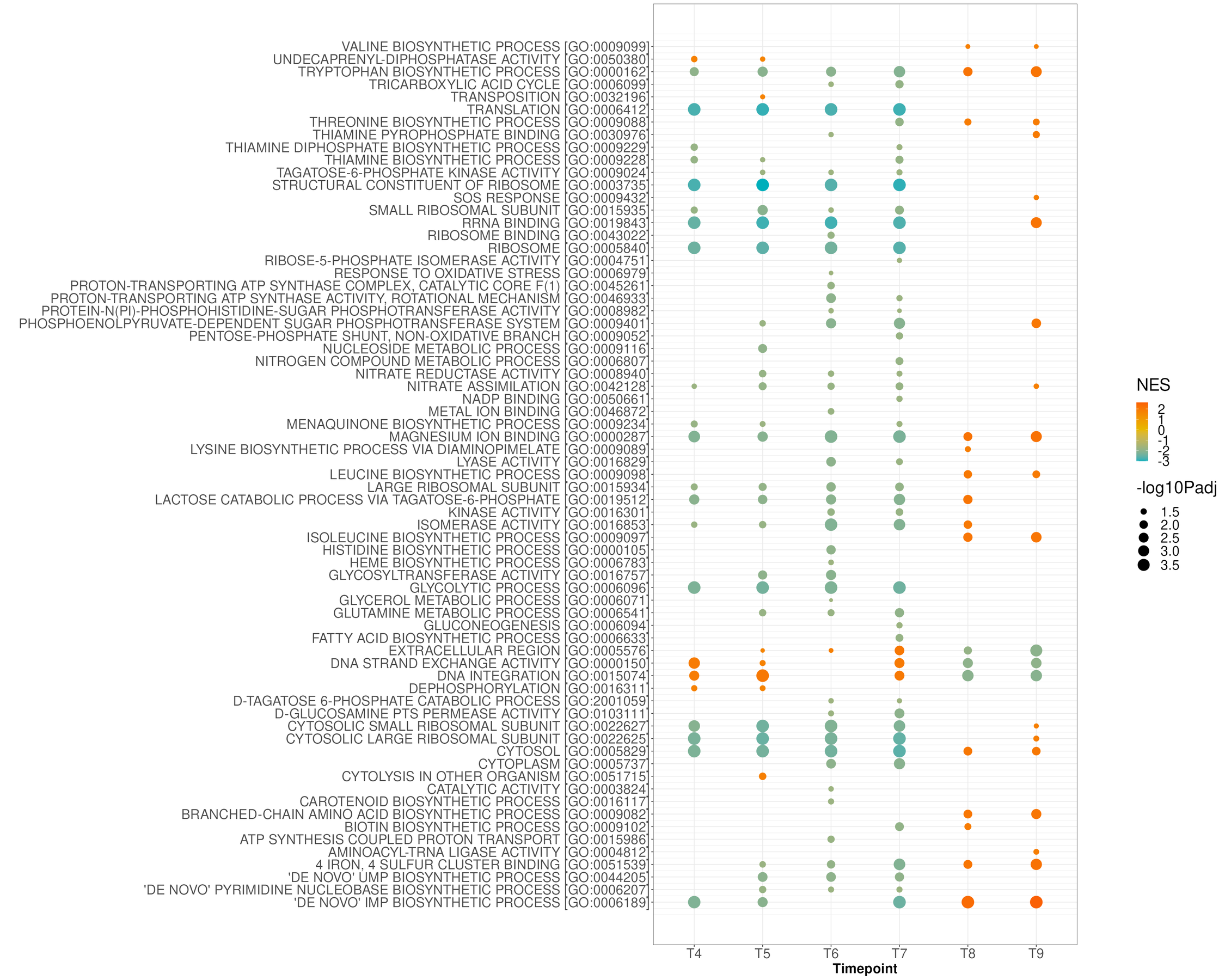

Supplement: S1 Fig — NES: Normalized Enrichment Score. (TIF) [file pone.0288758.s001.tif]

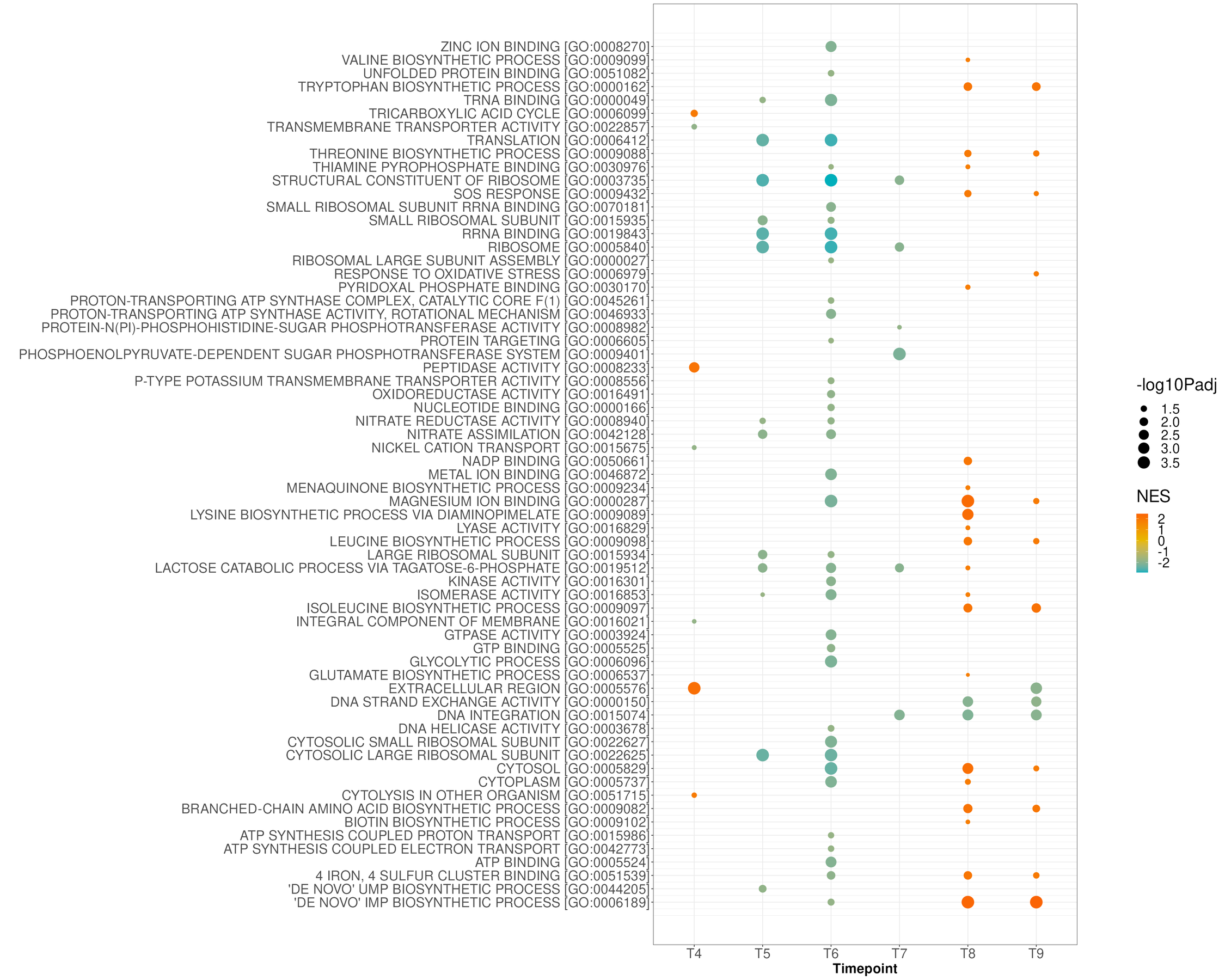

Supplement: S2 Fig — In the later stages, the most notable aspect was down-regulation of DNA Integration but up-regulation of several biosynthetic pathways most prominently amino-acids (leucine, tryptophan and threonine) of MRSA-9 versus MRSA-12. NES: Normalized Enrichment Score. (TIF) [file pone.0288758.s002.tif]

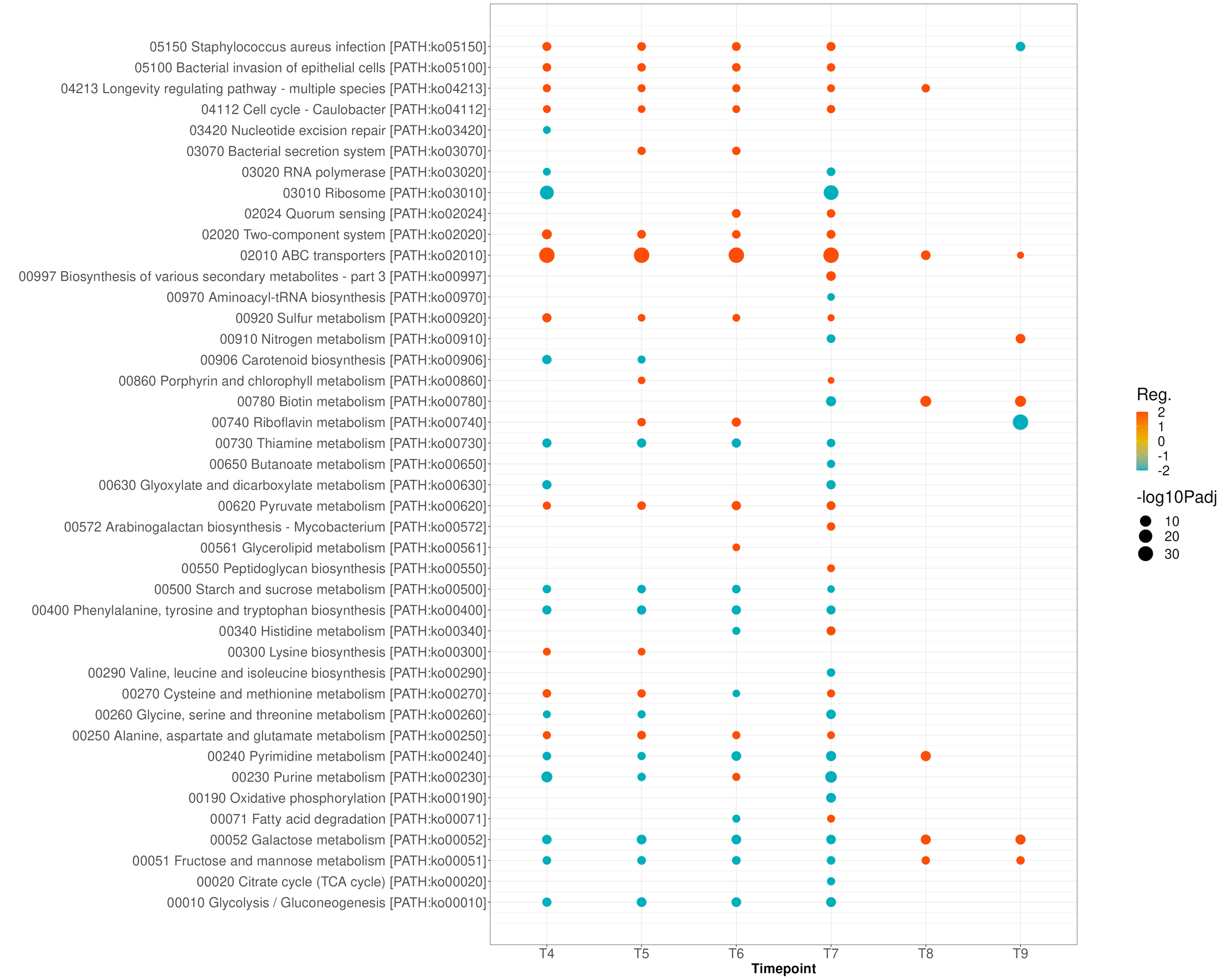

Supplement: S3 Fig — Notably the pathway’ ABC transporters’ was constantly upregulated throughout all time points. During the last 2 points, we notice a shift favoring metabolism and biosynthetic bring upregulated in MRSA-12 versus MSSA-29. KEGG: Kyoto Encyclopedia of Genes and Genomes. Reg.: Regulation. (TIF) [file pone.0288758.s003.tif]

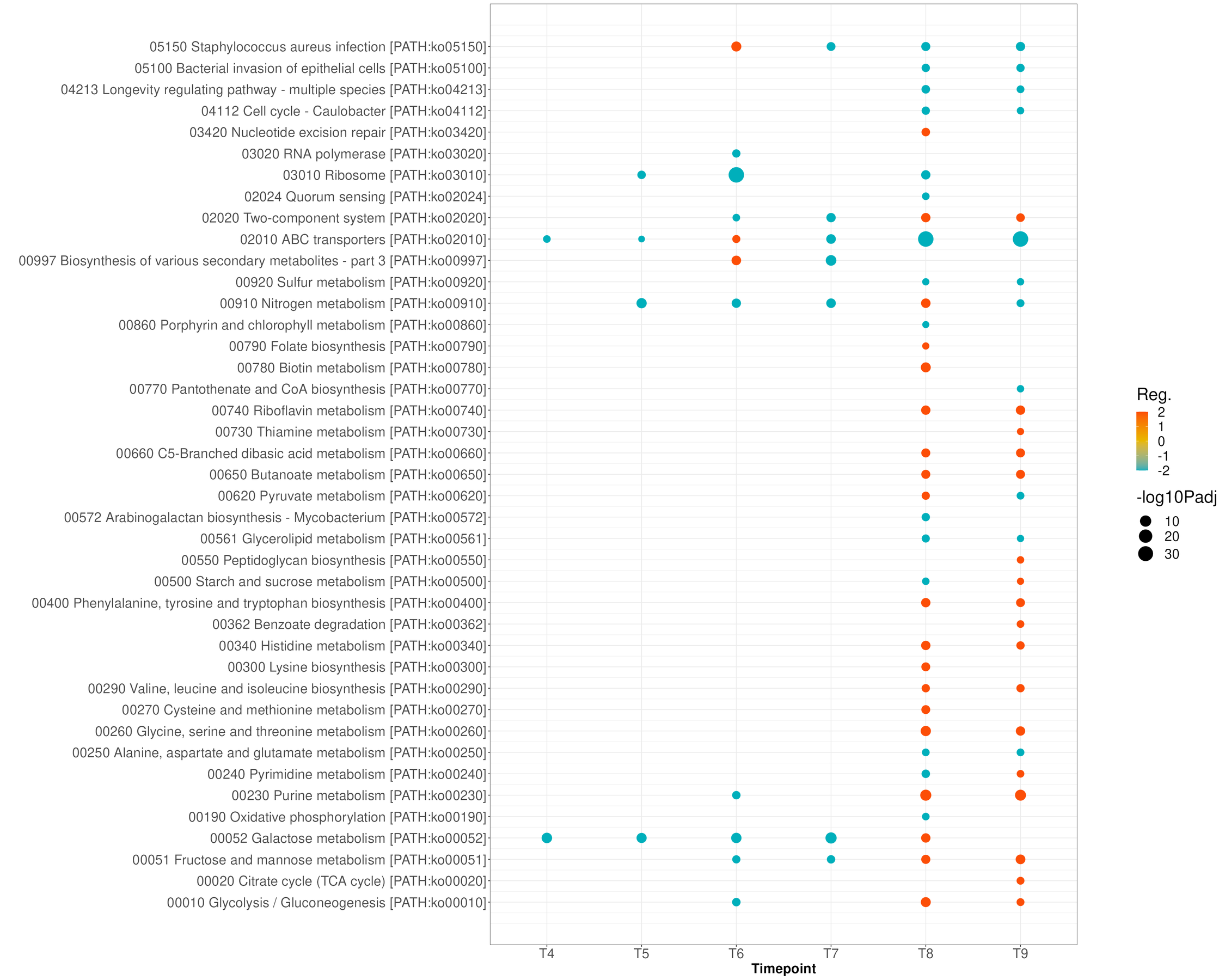

Supplement: S4 Fig — During the last 2 time points, a substantial shift in behavior with up-regulation of most metabolic and biosynthetic pathways and down-regulation of virulence pathways (ABC transporters, SA infection, Bacterial epithelial cells invasion) of MRSA-9 versus MRSA-12. KEGG: Kyoto Encyclopedia of Genes and Genomes. Reg.: Regulation. (TIF) [file pone.0288758.s004.tif]
