# Supplementary material for: Staphylococcus aureus isolates from children with clinically differentiated osteomyelitis exhibit distinct transcriptomic signatures
Source: PLoS One. 2023 Aug 10;18(8):e0288758. doi: 10.1371/journal.pone.0288758 (PMC10414669; doi:10.1371/journal.pone.0288758)
Supplement: S1 File — (DOCX) [file pone.0288758.s005.docx]

**PLOS-ONE Manuscript Reference #: PONE-D-23-00393**

**RNA Extraction – Step by Step Protocol**

Three isolates of *Staphylococcus aureus* were obtained from children with Acute Hematogenous Osteomyelitis (AHO) who had mild, moderate, and severe illness (MSSA29 – mild; MRSA12 – moderate; MRSA9 – severe). Total RNA sequencing was conducted for the isolates at six timepoints during the bacterial logarithmic growth phase. Bacterial RNA was isolated from 1.0 mL aliquots of broth culture obtained at 40-minute intervals from 4.5 to 7.8 hours. The selection of timepoints is described in greater detail in the Methods section of the manuscript. Each isolate was analyzed in triplicate by selecting separate single colonies for each culture preparation. This protocol describes the RNA isolation procedure that was performed once optimal culture growth was obtained. The extraction protocol was further optimized by performing both chemical and mechanical disruption of the bacterial cell wall, using lysostaphin and bead lysis respectively.

**Materials:**

1. RNeasy Mini Kit (Qiagen)
2. Buffer RPE (Qiagen)
3. Buffer RWT (Qiagen)
4. Qiazol Lysis Reagent (Qiagen)
5. Proteinase K (Qiagen)
6. Zymo RNA Clean and Concentrator-5 (Zymo)
7. Red RINO RNA Lysis Kit (Next Advance)
8. Turbo DNA free kit-Turbo Dnase, 10x Turbo Dnase buffer, Dnase Inactivation Reagent (Fisher Scientific)
9. Tris EDTA (TE) Buffer (Sigma)
10. Lysostaphin (Sigma)

**Preparation of reagents**

1. Lysostaphin: Reconstitute 1mg of lysostaphin in 1mL TE buffer. Store at -20C
2. TE buffer containing 100µg/ml lysostaphin (TE+Lysostaphin): Prepare sufficient volume for each sample to receive 200µl TE buffer containing lysostaphin (100 µg/mL)

**Procedure**

**Part A: Pellet bacterial culture**

1. Add 1 mL of the bacterial culture to RNA-free 1.5mL tubes
2. Centrifuge for 3 minutes at 13,300 RPM
3. Decant the supernatant. Remove remaining supernatant by gently dabbing the inverted tube once onto a paper towel. Pipette off as much broth as possible.
4. Place pellets on ice. Obtain pellets for all the logarithmic growth timepoints before starting Part B.

**Part B: Extraction by lysostaphin and mechanical disruption**

1. Add 20µl Proteinase K to 200µl TE buffer containing lysostaphin (described in ‘Preparation of reagents’ section above)
2. Add the 220µl mixture to the pellet. Carefully resuspend the pellet by pipetting up and down several times and vortex for 10 seconds.
3. Incubate at room temperature for 10 minutes. During the incubation, vortex for 10 seconds every 2 minutes.
4. Add 700µl of Qiazol to the resuspended cell pellet, mix up and down with pipette.
5. Transfer suspension into the RINO bead tube (Next Advance, Red RINO RNA Lysis Kit).
6. Homogenize for 1 minute at maximum speed (Next Advance BBY24M Bullet Blenders®). Place on ice for 1 minute.
7. Transfer solution by pipette to clean RNA-free 1.5mL tubes, avoid drawing up the beads in the tube.
8. Add 140µl chloroform to each tube. Close tube tops and vigorously shake tube 15 times.
9. The tubes remain at room temperature for 2 minutes, followed by 10-minute centrifugation at 4C, 13,300 RPM.
10. Transfer the supernatant (colorless, aqueous phase containing isolated RNA) to clean RNA-free 1.5mL tube.
11. Add 525 µl ethanol to each tube and mix by pipetting solution up and down.

**Part C: Buffer Wash**

1. Transfer exactly 700ul of the solution from Part B, step 15 to spin column placed in 2ml collection tube (Qiagen RNeasy Mini Kit).
2. Centrifuge for 30 seconds at 13,300 RPM. Discard the flowthrough in the spin column collection tube.
3. Add 700µl Buffer RWT (Qiagen) to the spin column, using the same collection tube.
4. Centrifuge for 30 seconds at 13,300 RPM. Discard flowthrough in the spin column collection tube.
5. Place the spin column into new collection tube from the Qiagen kit.
6. Add 500µl Buffer RPE (Qiagen) to the spin column.
7. Centrifuge for 30 seconds at 13,300 RPM. Discard the flowthrough in the spin column collection tube.
8. Add 500µl Buffer RPE (Qiagen) to spin column.
9. Centrifuge for 2 minutes at 13,300 RPM. Discard the flowthrough in the spin column collection tube.
10. Centrifuge for an additional 1 minute at 13,300 RPM to dry the column. Discard the flowthrough in the spin column collection tube.
11. Place column into new RNase free 1.5 ml tube.
12. Add 50µl of RNase-free water directly into the column. Close the column tube and centrifuge with the 1.5 ml tube open top. Spin for 1 min at 13,300 RPM. Be careful not to touch the column membrane.
13. Discard the pink column tube. Keep only the 1.5 ml tube. This tube contains the isolated RNA. The RNA is collected in this tube after the elution with RNase-free water from step 27.
14. OPTIONAL: Stopping point - At this step, the 1.5 ml tube containing the RNA can be stored at -80 until next day. No need to add a protect agent.

**Part D: DNase Treatment**

1. To each sample tube containing the isolated RNA solution, add 3µl 10X TURBO and 2µl of DNase (Fisher Scientific) and mix by gently flicking the tube.
2. Incubate tubes for 30 minutes at 37^◦^C.
3. Add 6µl of DNase Inactivation reagent to each tube.
4. Incubate tubes for 5 minutes at room temperature, mixing occasionally by gently flicking to re-dispense the DNase Inactivation Reagent.
5. Centrifuge at 10,000 RPM for 1.5 minutes and then transfer solution to new RNase free 1.5 ml tube. Leave the white precipitate at the bottom of the tube when transferring.

**Part E: RNA Elution using Zymo clean and concentrator**

1. To each sample tube, add 100 µl RNA binding buffer (from the Zymo clean and concentrator kit) and vortex.
2. Next add 150 µl pure ethanol and vortex.
3. Transfer the mixture from the 1.5 ml tube to the Zymo column (clear spin column tube that is placed in a collection tube) and centrifuge for 30 seconds at 12000 RPM. Discard flowthrough from the collection tube.
4. Add 400 µl RNA prep buffer (from to the spin column (same collection tube) and centrifuge for 30 sec at 12000 rpm. Discard flowthrough from the collection tube.
5. Wash the column with 700 µl RNA wash buffer (from the Zymo clean and concentrator kit) and centrifuge for 2 minutes at 12000 rpm. Discard flowthrough from the collection tube.
6. Centrifuge for 1 min to remove the wash buffer. Discard the flowthrough from collection tube.
7. Discard collection tube and place column into a new RNase free 1.5 ml tube.
8. Elute with 10 µl RNase free water onto the column that is placed in the new tube.
9. Centrifuge for 1 min (13.3 RPM). Keep the flowthrough in the 1.5 ml collection tube, this contains the final isolated RNA.
10. Add 20ul of RNase free water to 1.5 ml tube, mix gently by pipetting up and down.
11. Samples are now ready for quality and quantity analysis. Our laboratory utilized the Agilent Bioanalyzer 2100 and an RNA integrity number (RIN) > 9.0 was considered as good quality pass to proceed for sequencing.
